# Usefulness of Magnetoinertial Wearable Devices in Neurorehabilitation of Children with Cerebral Palsy

**DOI:** 10.1155/2018/5405680

**Published:** 2018-09-04

**Authors:** Marco Iosa, Manuela de Sanctis, Aurora Summa, Elena Bergamini, Daniela Morelli, Giuseppe Vannozzi

**Affiliations:** ^1^Clinical Laboratory of Experimental Neurorehabilitation, IRCCS Fondazione Santa Lucia, Via Ardeatina 306, 00179 Rome, Italy; ^2^Interuniversity Centre of Bioengineering of the Human Neuromusculoskeletal System, Department of Movement, Human, and Health Sciences, University of Rome “Foro Italico”, Piazza Lauro De Bosis 15, 00135 Rome, Italy

## Abstract

**Background:**

Despite the increasing use of wearable magnetoinertial measurement units (MIMUs) for gait analysis, the efficacy of MIMU-based assessment for planning rehabilitation has not been adequately documented yet.

**Methods:**

The usefulness of a MIMU-based assessment was evaluated comparing the data acquired by three MIMUs located at the pelvis, sternum, and head levels in 12 children with cerebral palsy (CP, age: 2–9 years) and 12 age-matched children with typical development (TD). Gait stability was quantified in terms of acceleration attenuation coefficients from pelvis to head, pelvis to sternum, and sternum to head. Children with CP were randomly divided in two groups: in the first group (CPI), MIMU-based parameters were used by therapists for planning patient-tailored rehabilitation programs, whereas in the second group (CPB), therapists were blind to the MIMU-based assessment results. Both CPI and CPB were tested before and after the relevant neurorehabilitation program. Ad hoc questionnaires were also administered to therapists of the CPI group to assess the degree of usefulness perceived about the information provided by the MIMU-based assessment.

**Results:**

Significant differences were found between children with CP and those with TD for the acceleration attenuation coefficient from pelvis to head (*p* = 0.048) and from pelvis to sternum (*p* = 0.021). After neurorehabilitation, this last parameter increased more in CPI (35%) than in CPB (6%, *p* = 0.017 for the interaction group per time). The results of the questionnaires showed that therapists agreed with the usability (100% judged it as “easy to use”) and usefulness of the MIMU-based assessment in defining patient-oriented interventions (87%).

**Conclusions:**

There is a large debate in literature about the efficacy of classical gait analysis that should be enlarged to new technological approaches, such as that based on MIMUs. This study is a first proof of concept about the efficacy of this approach for neurorehabilitation of children with CP.

## 1. Introduction

Since the introduction of instrumented stereophotogrammetric analysis of human walking in clinical settings, it has been debated whether gait analysis can effectively provide information about functional deficits that is useful for the clinical management of patients as well as for assessing the outcomes of a therapeutic intervention [1–6].

More recently, there has been an increasing interest in the use of wearable devices based on MIMU technology (MIMU: magnetoinertial measurement unit) for gait analysis [7]. Potential benefits of wearable inertial devices include their low cost with respect to stereophotogrammetric systems, commonly used for clinical gait analysis; their small dimensions and light weight; the absence of limitation of the testing environment to a laboratory; the fact that they are easy to apply on patients; and their user-friendliness. All these characteristics make them ideal for being used in the clinical context [7–9].

MIMU technology for gait analysis has been used to assess gait stability [7] by means of a single unit located on the trunk [10–13]. The use of a single MIMU, however, does not allow obtaining information about the role of the whole trunk and head, which, in patients with neurological disorders, may be crucial both in movement control and postural balance [14]. In this respect, acceleration data, measured at different body levels in the three anatomical directions, can provide insightful information about upright gait stability [15–18], which, can be also described as the capacity to minimize oscillations during walking, in a progressive way, from the lower to the upper levels of the human body [19]. Healthy subjects typically present a progressive reduction of the acceleration from pelvis to sternum and from sternum to head, that reflects the adoption of postural control strategies leading to a steady visual input and to a more effective processing of the vestibular system signals, thus improving the control of equilibrium [20]. This control is developed early in children, progressively lost with aging, and altered in the presence of a neurological deficit [15, 16, 18, 21, 22].

Given all the abovementioned benefits characterizing the use of MIMUs in clinics, children with cerebral palsy (CP) are among the best candidates for being assessed with this approach [17, 18]. The assessment of upright gait stability in these children suggested that their altered upper-body stability during walking is the result of a trunk control deficit as well as of movements performed for compensating lower limb impairments [23]. This is reflected by increased ranges of motion of head and trunk segments in each anatomical plane [24, 25], leading to difficulty in attenuating the existing high accelerations from lower- to upper-body levels with respect to children with typical development (TD) [18].

Despite this evidence, in the literature, a lack exists concerning the evaluation of the actual efficacy of MIMU-based assessment protocols in the clinical context [8, 9]. Specifically, no evidence exists about the clinical usefulness of the abovementioned MIMU-based parameters related to upper-body gait stability, that is, can these parameters effectively support health professionals in planning patient-specific rehabilitation programs? What is the actual perception of therapists about MIMU-based assessment protocols' usability and usefulness?

The aim of this study was to answer these questions and, in particular, to evaluate if a MIMU-based assessment of upper-body accelerations in children with CP represents an actual added value for planning patient-specific intervention. To this aim, MIMU-based gait stability parameters were obtained for a group of children with CP and first compared to the values obtained for a group of children with typical development. Second, the estimated parameters were used by therapists for planning individualized neurorehabilitation programs. The effects of these programs were assessed on half of the group, whereas the other half performed standard rehabilitation. The degree of usefulness perceived by therapists about the information provided by the MIMU-based assessment was also evaluated by means of ad hoc questionnaires.

## 2. Material and Methods

### 2.1. Study Design

The ability of a MIMU-based assessment to discriminate between children with CP and children with typical development was already demonstrated in previous studies [17, 18]. This aspect has been dealt with also in the present study and two main innovative aspects were considered: the test of the sensitivity, specificity, and accuracy of the MIMU-based analysis and the usefulness of this assessment for designing children rehabilitation interventions. Children with CP were randomly divided in two groups: in the first group (CPI, I: informed) MIMU-based parameters were used by therapists for planning a patient-tailored rehabilitation program, whereas in the second group (CPB, B: blind), therapists were blind to the MIMU-based assessment results. All patients were assessed at the beginning (T0) and at the end (T1) of a six-month period of neurorehabilitation program. inally, two questionnaires were administered to the therapists of the CPI group to investigate the degree of usefulness of the MIMU assessment in the therapeutic management of children.

### 2.2. Participants

Twelve children with clinical diagnosis of cerebral palsy (CP) and twelve children with typical development (TD) were recruited (CP: 5.70 ± 2.27 years, range 2–9 years, TD: 5.42 ± 2.01 years, range 2–9 years). Both groups were matched for gender and age, and were characterized by similar stature (CP: 1.10 ± 0.18 m, TD: 1.14 ± 0.15 m) as well as similar leg length (defined as the distance between the greater trochanter and the lateral malleolus measured while standing, CP: 0.54 ± 0.11 m, TD: 0.54 ± 0.09 m). Both groups were formed by 7 females and 5 males. The CP group included 7 subjects with hemiparesis and 5 with diplegia. All children with TD involved in the study were physically active and had no neurological, orthopaedic, or motor disorder.

The experimental protocols were approved by the Ethics Committee of the Santa Lucia Foundation. All participants gave their assent and either their parents or legal guardians provided written informed consent according to the declaration of Helsinki. The whole group was classified by expert physiotherapists according to the gross motor function classification system (GMFCS) [26], obtaining an average score of 1.75 ± 0.75. A group of 7 physical therapists treating the 6 children of CPI was involved (mean age: 32.0 ± 1.2 years, mean professional experience: 8.5 ± 0.6 years, the ratio was 1 therapist for each child, with the exception of 1 child alternatively treated by 2 therapists, both informed by the results of a MIMU test and involved in the group of therapists answering to the questionnaires).

### 2.3. Assessment Protocol

**Figure 1:**
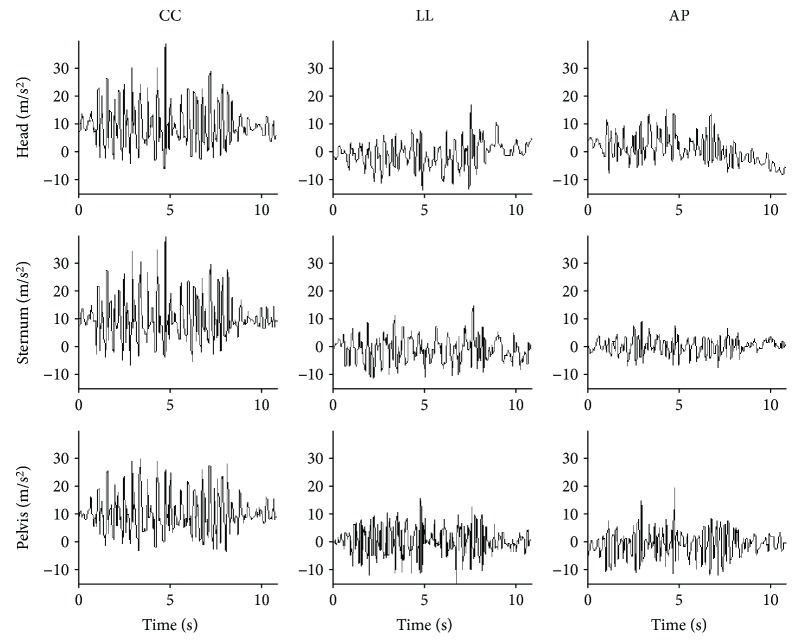
Raw acceleration data for a randomly chosen child with TD.

Children were asked to maintain a standing position for about 3 s and, then, to perform a 10-meter walking test on a linear pathway at their self-selected walking speed looking ahead. The start and the stop lines of the pathway were marked by two visible strips. Each subject performed three trials. Video recordings were collected for each trial using a commercial video camera (JVC GC-PX10 HD Memory) to control trial performance during data postprocessing. Children performed the walking test wearing three magnetoinertial measurement units (MIMUs, Opal, APDM Inc., Oregon, USA) which were used to collect 3D acceleration, angular velocity, and magnetic field vector components [18]. Sample rate was set to 128 samples/s and full-range scale was set to ±2 g (*g* = 9.81 m/s^2^) for accelerometers, ±1500 °/s for gyroscopes, and ±600 *μ*T for magnetometers. Children were dressed with adjustable supports including swim caps, stretch tops, and stretch shorts, each one having pockets tailored for housing the MIMUs. The three units were positioned, respectively, at head level (H), on the occipital cranium bone, at the center of the sternum (S), and at sacrum-L5 level on the pelvis (P). The three MIMU orthogonal axes were carefully aligned to the craniocaudal (CC), mediolateral (ML), and anteroposterior (AP) axes of each body segment.  Figure 1 shows raw acceleration data for a randomly chosen child with TD for the units located on the pelvis, sternum, and head along CC, ML, and AP axes.

### 2.4. MIMU Data Analysis

Data were processed using custom algorithms implemented in MATLAB® (The MathWorks Inc., MA, US). The inclination of each MIMU with respect to gravity was computed during the initial part of the test, when each participant was stationary in a standing position. To guarantee a repeatable system of reference for all participants, each MIMU was then rotated following the pitch-roll-yaw rotation sequence so as to have one axis aligned with gravity during the abovementioned stationary phase [27]. Then, for each MIMU, a local anatomical frame was defined to match the rotated MIMU reference frame and was characterized by the CC, AP, and ML axes. The CC axis of each local frame was aligned with gravity only during the stationary phases at the beginning of each trial. The gravitational acceleration component was removed from the measured acceleration signals using the orientation data provided by the MIMUs' proprietary algorithm. The acceleration components, recorded during walking, were low-pass filtered using a 4th-order Butterworth filter. The cutoff frequency was determined by performing a residual analysis [28] on each acceleration component. As the values obtained were similar among different components, trials, and participants, the cutoff frequency value was conservatively set to 20 Hz for all trials. The accelerations were then expressed in the relevant local anatomical frame.

The time taken to complete the 10-meter walking test and the foot strike events were obtained by identifying consistent features (maxima) on the pelvis acceleration time histories using the methodology described in [29] and by comparing this information with video data. From this information, both step frequency (number of steps/time (s^−1^)) and average step length (total distance/number of steps (m)) were estimated. Only the central part of the pathway was considered for further data processing. In particular, steady-state walking phases were identified as in [30] and, for each trial, two consecutive strides (four steps) were selected within these phases and further considered for the analysis to reduce the effect of the walking speed variations on the AP acceleration measures. The average walking speed (m·s^−1^) was computed as the ratio between the average length of the two analyzed strides and the relevant duration. To account for the influence of participants' stature on average step length, step frequency, and walking speed, the corresponding normalized parameters were computed [31]. The average of the values obtained on the three tests was further considered for each subject.

The root mean square of the accelerations (RMS*_a_*) were then computed at the three levels of the upper body for each stride, using both the magnitude of the acceleration as well as each component as expressed in the local anatomical frame. The variation of the RMS*_a_* from level *i* to upper level *j* of the upper body was assessed by computing the relevant attenuation coefficients (*C_PS_*, *C_SH_*, and *C_PH_*) as follows [32]:

(1) $$\begin{array}{c} C_{ij}=\left(1-\frac{{RMS}_{aj}}{{RMS}_{ai}}\right)\cdot 100. \end{array}$$

Average values of these parameters over different strides, trials, and participants were then obtained.

### 2.5. Questionnaires for Therapists

As reported in Section 2.1, children and the relevant therapists treating them (the ratio was one therapist for each child) were randomly divided in two groups: CPI and CPB. The six therapists treating CPI were informed about the results of the MIMU-based assessment, whereas the six ones treating CPB remained blind to this information.

To assess the degree of usefulness perceived by therapists about the information provided by the MIMU-based assessment, two questionnaires administered to the therapists of CPI were assessed. Both questionnaires are based on a Likert scale using five points (completely disagree, disagree, neither agree nor disagree, agree, and completely agree). The first was administered at T0 and was formed by 4 questions. This questionnaire examined whether the MIMU-based assessment was (1) easy, (2) in line with clinical observation, (3) more informative than clinical observation, and (4) useful for designing therapeutic programs. The second questionnaire was administered at T1 and formed by 3 other questions. The first two questions, with answers based on the above Likert scores, investigated (1) if the information given by the MIMU-based assessment was useful during the planning of the treatment and (2) if this information influenced the rehabilitative decision-making process. The last yes/no question was included where therapists had to say if they would like to have the results of the MIMU-based assessments along the rehabilitation program of other children.

### 2.6. Statistical Analysis

The statistical analysis was performed using the IBM SPSS Statistics software package version 23 (IBM Corp., Armonk, NY, USA). A mixed analysis of variance (ANOVA) was first used to compare the coefficients of attenuation between children with CP (at T0) and those with TD (group factor), among the different axes (within-subject factor), computing *F* value, *p* value, and the effect size evaluated in terms of partial eta squared (ES). A two-step cluster analysis based on log-likelihood distance algorithm (blind to the sample size of each group) was used to identify subjects with lower versus those with higher attenuation of acceleration, based on the nine coefficients of attenuation (pelvis to head, pelvis to shoulder, and shoulder to head per the three body axes). From the obtained categorization, sensitivity was computed as the percentage of true positives (children with CP classified as those having lower coefficients) divided by the entire number of positives (true positives plus false negatives). Analogously, specificity was computed as the percentage of true negatives (children with TD classified as those having higher coefficients) divided by the entire number of negatives (true negatives plus false positives). Finally, accuracy was computed as the percentage number of true positives plus true negatives on the total number of cases. Then, the attenuation coefficient values obtained by CPI versus CPB (group factor) between T0 and T1 (time within-subject factor) along each axis (within-subject factor) were compared using the same abovementioned mixed ANOVA. For the questionnaires, descriptive statistics was obtained. The alpha level of significance was set to 0.05 for all statistical tests.

## 3. Results and Discussion

### 3.1. Comparisons between Children with CP and Children with TD

**Figure 2:**
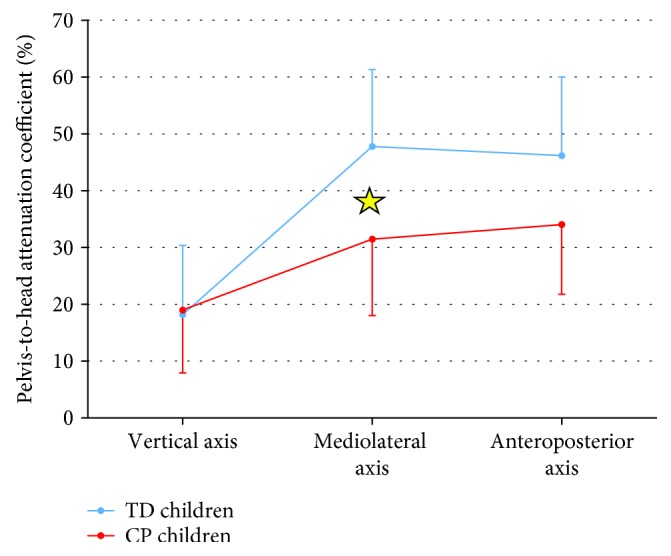
Mean and standard deviation for the pelvis-to-head coefficient of acceleration for children with CP (black) and for those with TD (grey). The star indicates a significant difference obtained with post hoc analysis.

Figure 2 shows the differences between the CP (at T0) and TD groups in terms of the pelvis-to-head coefficient of attenuation. This coefficient resulted significantly different between the two groups (*F* = 4.383, *p* = 0.048, ES = 0.166). The significant differences among the three body axes (*F* = 24.807, *p* < 0.001, ES = 0.530) resulted significant also with respect to the two groups (interaction group^∗^axis: *F* = 3.271, *p* = 0.047, ES = 0.129). This last interaction was statistically significantly also for the attenuation coefficient between the pelvis and sternum (*F* = 4.224, *p* = 0.021, ES = 0.161). In addition, the attenuation coefficient between the sternum and head resulted significantly different between the two groups (*F* = 20.484, *p* < 0.001, ES = 0.482).

These results confirmed the existing literature about the differences between children with CP with respect to age-matched children with TD, assessed using one [17] or multiple [18] inertial sensor devices. Although informative about describing the functional limitations characterizing children with CP with respect to those with TD, these differences are not sufficient to prove the actual usefulness of a MIMU-based approach in neurorehabilitation. For this reason, we included the cluster analysis, the assessment of changes after therapy, and also the analysis of therapists' answers to questionnaires.

The cluster analysis based on the coefficients of attenuation identified nine children with lower coefficients, all of them affected by CP. Three children with CP were not identified by the algorithm as positives. Hence, the sensitivity of the MIMU approach was 75%. All children with TD were identified as negatives, so the specificity of the approach was 100%. The resultant accuracy was 87.5%. These findings represent a step forward with respect to previous studies [17, 18] in which the differences highlighted by the MIMU approach resulted statistically significant between groups, but no evidence was provided about their sensitivity nor specificity. Our results showed good accuracy, sensitivity, and especially specificity of attenuation coefficients in identifying patients' deficits, even using an algorithm blind to the number of subjects that should be classified as pathological.

### 3.2. Comparisons between the Two Groups of Children with CP

The following analyses aimed at assessing the changes pre- and postrehabilitation (between T0 and T1) in the two groups of children with CP. It is noteworthy that the analysis of walking speed was not sufficient to highlight changes between pre- and postrehabilitation (*F* = 0.675, *p* = 0.430, ES = 0.063), nor between the two groups of children with CP (*F* = 0.326, *p* = 0.581, ES = 0.032). Similarly, the interaction of these two factors on walking speed was not statistically significant (*F* = 2.304, *p* = 0.160, ES = 0.187). This is an important aspect, showing that for children with cerebral palsy the assessment of walking speed, that could be easily performed by means of clinical walking tests (such as the ten-meter walking test or the six-minute walking test), is often not sufficient for having a clinical picture of children's deambulation ability [17]. The clinical relevance of upright gait stability parameters is an important issue that deserves attention. A previous work of our research group [16] highlighted the importance of an instrumental MIMU assessment test in a sample of 45 patients with stroke, showing significant correlation with clinical scale scores. The change in pelvic-to-sternum accelerations differed significantly among the subgroups of patients divided according to ambulation ability, being able to identify patients at risk of fall.

In summary, these instrumental indices, especially the pelvis-to-sternum acceleration attenuation, provide valuable information about the different motor strategies implemented by each patient during walking for maintaining dynamic balance, complementing and integrating the outcomes of traditional clinical scales, for limiting the risk of fall (in adults) and to design specific treatments (for adults and children).

Conversely, the trunk-to-sternum attenuation coefficient increased 35% in CPI and increased 6% in CPB, from T0 to T1.  Table 1 shows the results of the mixed ANOVA for this parameter, between CPI and CPB, between T0 and T1, and for the three anatomical axes. The significant interaction between “group” and “time” showed that the increase of 35% found for CPI was significantly higher than the 6% found in CPB. On the other hand, the coefficient of attenuation from pelvis to head did not show any statistically significant differences, most probably due to the high variability of head accelerations.

Previous studies showed that children with CP have large accelerations at the pelvis level, suggesting that this is an important clinical parameter at the basis of the reduced gait stability in this population [17, 18]. It has been argued that patients affected by CP, even if displaying an increased pelvis-to-sternum attenuation, do not adequately reduce high acceleration values at the pelvis level, exhibiting accelerations at the sternum level which are still greater than in the TD counterpart [18].

The importance of our findings relies on the proof of concept that, not only can MIMU-based parameters be used by therapists to design specific patient-tailored interventions, but also relying on these parameters when designing individualized rehabilitation programs has a significant impact on program efficacy. To corroborate these findings, more extensive research is required to investigate the potential benefits of MIMU-based approaches on larger samples of children with CP, possibly divided with respect to the pathology severity (GMFCS levels), the affected side (hemiplegia versus diplegia), as well as the gender/age.

A limit of our study is related to the reduced sample size that probably led to the absence of significant differences in terms of pelvis-to-head accelerations. It must be mentioned that, despite the request for children to walk looking forward, this parameter can be strongly affected even by small head rotations due to distractions. In this respect, this aspect should deserve special attention during MIMU-based assessments when the head is involved in the measurements. Despite that measuring accelerations at the head level could be important for having information about the stabilization of vestibular and visual signals, both used for improving balance [20], these measures seemed to be too much sensitive to voluntary movements in the children enrolled in our study.

### 3.3. Results of Questionnaires Administered to Therapists

After the first MIMU-based assessment (T0), four therapists declared to agree (57%) and three completely agreed (43%) with the fact that the test was easily performed. Four (57%) therapists declared to agree (3, 43%) or completely agreed (1, 14%) that the results of the MIMU assessment were in line with clinical observations (the remaining three therapists, 43%, neither agreed nor disagreed). An important result is that five (71%) therapists agreed and two (29%) completely agreed that the tests provided more information than clinical observation alone. Four therapists declared to agree (57%) and three completely agreed (43%) with the usability of the received information for designing rehabilitation interventions.

After six months of rehabilitation, the usefulness of the information was confirmed by therapists (four agreed, 57%, two completely agreed, 29%, and one neither agreed nor disagreed, 14%). Six (86%) therapists agreed with the fact that they defined the rehabilitation program in accordance with the information given by the MIMU-based assessment (the remaining therapist neither agreed nor disagreed). Five therapists (71%) declared not to have found any difficulties in using MIMU-based information for planning the rehabilitation program (whereas the other two therapists found some difficulties). Finally, all seven therapists (100%) declared that they would also benefit from MIMU-based assessments in the future, for other children.

The results of these structured questionnaires confirm the usefulness of the MIMU-based approach as perceived by therapists and as proven by its actual use during rehabilitation. These findings confirmed that the MIMU-based assessment was perceived by therapists as easy-to-use and providing useful information for designing rehabilitation programs. Indeed, it allows for a more specific patient-tailored therapy that is a fundamental aspect, especially for children, considering that the changes of their disability are strictly interconnected with their development.

## 4. Conclusions

The present work focused on the application of the MIMU-based assessment in a real clinical setting, involving children with CP and their therapists. The results of this study support the use of MIMU-based technology for the assessment of gait stability in children with CP (good accuracy, sensitivity, and especially specificity) and prove that MIMU-based parameters not only provide valuable information for designing patient-specific rehabilitation programs but can also represent an actual added value in terms of these programs' efficacy. Furthermore, the usability of this approach was confirmed by therapists, who agreed on its user-friendliness and high information content.

This study provided, therefore, a first proof of concept about the possibility of an actual useful application of MIMUs for guiding patient-tailored neurorehabilitation interventions designed on the basis of quantitative objective information.

## Figures and Tables

**Table 1:** Results of mixed ANOVA on the pelvis-sternum coefficient of attenuation (*F* value, *p* value, and ES: effect size).

Factor	*F*	*P*	ES
Group (CPI versus CPB)	0.035	0.855	0.033
Time (pre- versus postrehab)	3.524	0.090	0.261
Axis (AP versus ML versus CC)	9.088	**0.002**	0.476
Interaction group × time	8.211	**0.017**	0.451
Interaction group × axis	7.805	**0.003**	0.483
Interaction time × axis	0.105	0.901	0.010
Interaction group × time × axis	3.139	0.065	0.239

## Data Availability

The data sets used and/or analyzed in the current study are available from the corresponding author upon reasonable request.

## References

[1] Gage J. R., Fabian D., Hicks R., Tashman S. (1984). Pre- and postoperative gait analysis in patients with spastic diplegia: a preliminary report. *Journal of Pediatric Orthopaedics*.

[2] Follak N., Ganzer D., Merk H. (2002). The utility of gait analysis in the rehabilitation of patients after surgical treatment of Achilles tendon rupture. *European Journal of Orthopaedic Surgery and Traumatology*.

[3] Watts H. G. (1994). Editorial. Gait laboratory analysis for preoperative decision making in spastic cerebral palsy: is it all it’s cracked up to be?. *Journal of Pediatric Orthopaedics*.

[4] Morgan D., Funk M., Crossley M., Basran J., Kirk A., Bello-Haas V. D. (2007). The potential of gait analysis to contribute to differential diagnosis of early stage dementia: current research and future directions. *Canadian Journal on Aging/La Revue canadienne du vieillissement*.

[5] Cimolin V., Galli M. (2014). Summary measures for clinical gait analysis: a literature review. *Gait & Posture*.

[6] Benedetti M. G., Beghi E., de Tanti A. (2017). SIAMOC position paper on gait analysis in clinical practice: general requirements, methods and appropriateness. Results of an Italian consensus conference. *Gait & Posture*.

[7] Iosa M., Picerno P., Paolucci S., Morone G. (2016). Wearable inertial sensors for human movement analysis. *Expert Review of Medical Devices*.

[8] Vienne A., Barrois R. P., Buffat S., Ricard D., Vidal P. P. (2017). Inertial sensors to assess gait quality in patients with neurological disorders: a systematic review of technical and analytical challenges. *Frontiers in Psychology*.

[9] Wang Q., Markopoulos P., Yu B., Chen W., Timmermans A. (2017). Interactive wearable systems for upper body rehabilitation: a systematic review. *Journal of Neuroengineering and Rehabilitation*.

[10] Kavanagh J. J., Menz H. B. (2008). Accelerometry: a technique for quantifying movement patterns during walking. *Gait & Posture*.

[11] Bisi M. C., Riva F., Stagni R. (2014). Measures of gait stability: performance on adults and toddlers at the beginning of independent walking. *Journal of Neuroengineering and Rehabilitation*.

[12] Lowry K. A., Smiley-Oyen A. L., Carrel A. J., Kerr J. P. (2009). Walking stability using harmonic ratios in Parkinson’s disease. *Movement Disorders*.

[13] Riva F., Toebes M. J. P., Pijnappels M., Stagni R., van Dieën J. H. (2013). Estimating fall risk with inertial sensors using gait stability measures that do not require step detection. *Gait & Posture*.

[14] Isho T., Usuda S. (2016). Association of trunk control with mobility performance and accelerometry-based gait characteristics in hemiparetic patients with subacute stroke. *Gait & Posture*.

[15] Belluscio V., Bergamini E., Iosa M., Tramontano M., Morone G., Vannozzi G. (2018). The iFST: an instrumented version of the Fukuda Stepping Test for balance assessment. *Gait & Posture*.

[16] Bergamini E., Iosa M., Belluscio V., Morone G., Tramontano M., Vannozzi G. (2017). Multi-sensor assessment of dynamic balance during gait in patients with subacute stroke. *Journal of Biomechanics*.

[17] Iosa M., Marro T., Paolucci S., Morelli D. (2012). Stability and harmony of gait in children with cerebral palsy. *Research in Developmental Disabilities*.

[18] Summa A., Vannozzi G., Bergamini E., Iosa M., Morelli D., Cappozzo A. (2016). Multilevel upper body movement control during gait in children with cerebral palsy. *PLoS One*.

[19] Cappozzo A. (1982). Low frequency self-generated vibration during ambulation in normal men. *Journal of Biomechanics*.

[20] Berthoz A., Pozzo T., Swinnen S. P., Heuer H., Massion J., Casaer P. (1994). 7 - head and body coordination during locomotion and complex movements. *Interlimb Coordination*.

[21] Buckley C., Galna B., Rochester L., Mazzà C. (2015). Attenuation of upper body accelerations during gait: piloting an innovative assessment tool for Parkinson’s disease. *BioMed Research International*.

[22] Iosa M., Fusco A., Morone G., Paolucci S. (2014). Development and decline of upright gait stability. *Frontiers in Aging Neuroscience*.

[23] Heyrman L., Feys H., Molenaers G. (2014). Altered trunk movements during gait in children with spastic diplegia: compensatory or underlying trunk control deficit?. *Research in Developmental Disabilities*.

[24] Heyrman L., Feys H., Molenaers G. (2013). Three-dimensional head and trunk movement characteristics during gait in children with spastic diplegia. *Gait & Posture*.

[25] Romkes J., Peeters W., Oosterom A. M., Molenaar S., Bakels I., Brunner R. (2007). Evaluating upper body movements during gait in healthy children and children with diplegic cerebral palsy. *Journal of Pediatric Orthopaedics. Part B*.

[26] Palisano R., Rosenbaum P., Walter S., Russell D., Wood E., Galuppi B. (1997). Development and reliability of a system to classify gross motor function in children with cerebral palsy. *Developmental Medicine & Child Neurology*.

[27] Bergamini E., Ligorio G., Summa A., Vannozzi G., Cappozzo A., Sabatini A. (2014). Estimating orientation using magnetic and inertial sensors and different sensor fusion approaches: accuracy assessment in manual and locomotion tasks. *Sensors*.

[28] Winter D. A., Winter D. (1990). *Biomechanics and Motor Control of Human Movement*.

[29] Brandes M., Zijlstra W., Heikens S., van Lummel R., Rosenbaum D. (2006). Accelerometry based assessment of gait parameters in children. *Gait & Posture*.

[30] Masci I., Vannozzi G., Bergamini E., Pesce C., Getchell N., Cappozzo A. (2013). Assessing locomotor skills development in childhood using wearable inertial sensor devices: the running paradigm. *Gait & Posture*.

[31] Hof A. L. (1996). Scaling gait data to body size. *Gait & Posture*.

[32] Mazzà C., Iosa M., Pecoraro F., Cappozzo A. (2008). Control of the upper body accelerations in young and elderly women during level walking. *Journal of NeuroEngineering and Rehabilitation*.

